# Improving Dengue Diagnostics and Management Through Innovative Technology

**DOI:** 10.1007/s11908-018-0633-x

**Published:** 2018-06-07

**Authors:** Jesus Rodriguez-Manzano, Po Ying Chia, Tsin Wen Yeo, Alison Holmes, Pantelis Georgiou, Sophie Yacoub

**Affiliations:** 1 Centre for Bio-inspired Technology, Department of Electrical and Electronic Engineering, Imperial College London, London, UK; 2 Communicable Diseases Centre, Institute for Infectious Disease and Epidemiology, Tan Tock Seng Hospital, Singapore, Singapore; 3 Lee Kong Chian School of Medicine, Nanyang Technical University, Singapore, Singapore; 4 Department of Medicine, Imperial College London, London, UK; 5 Singapore-MIT Alliance for Research and Technology, Singapore, Singapore; 6 Oxford University Clinical Research Unit, Wellcome Trust Asia Programme, Ho Chi Minh City, Vietnam

**Keywords:** Dengue diagnostics, Management, Novel technology, Wearables, Sensors, Point-of-care

## Abstract

**Purpose of Review:**

Dengue continues to be a major global public health threat. Symptomatic infections can cause a spectrum of disease ranging from a mild febrile illness to severe and potentially life-threatening manifestations. Management relies on supportive treatment with careful fluid replacement. The purpose of this review is to define the unmet needs and challenges in current dengue diagnostics and patient monitoring and outline potential novel technologies to address these needs.

**Recent Findings:**

There have been recent advances in molecular and point-of-care (POC) diagnostics as well as technologies including wireless communication, low-power microelectronics, and wearable sensors that have opened up new possibilities for management, clinical monitoring, and real-time surveillance of dengue.

**Summary:**

Novel platforms utilizing innovative technologies for POC dengue diagnostics and wearable patient monitors have the potential to revolutionize dengue surveillance, outbreak response, and management at population and individual levels. Validation studies of these technologies are urgently required in dengue-endemic areas.

**Electronic supplementary material:**

The online version of this article (10.1007/s11908-018-0633-x) contains supplementary material, which is available to authorized users.

## Introduction

Dengue virus (DENV) has emerged in the last two decades as the largest arboviral threat to humans, with 390 million infections estimated to occur every year, of which 96 million are clinically apparent [1•]. This unprecedented increase in dengue cases has been driven by a number of factors, including an increase in international travel, globalization, climate change, and huge expansion of the geographical range of the vector, *Aedes* mosquitoes, coupled with urbanization, suboptimal urban planning, and limited public health interventions [2]. DENV is a flavivirus with four antigenically distinct serotypes (DENV-1 to DENV-4) [3]. Infection with one serotype provides lifelong serotype-specific immunity; however, secondary infection with a different serotype carries an increased risk of severe disease, due to a phenomenon known as antibody-dependent enhancement [4••]. There is abundant genetic variation within each serotype in the form of phylogenetically distinct clusters of sequences called subtypes or genotypes [5]. There is evidence that certain genotypes may be “fitter” and can differ in their capacity to cause severe disease [6, 7]. Modeling studies in endemic areas have shown the yearly seasonal fluctuations in incidence and epidemics are shaped by population immunity to heterologous dengue serotypes and serotype/genotype replacement, as well as vector capacity [8].

Due to the potential for more severe disease in sequential infections, developing a safe and balanced vaccine for all four serotypes has been challenging; however, in 2015, the first ever dengue vaccine was licensed; a tetravalent live attenuated vaccine (CYD-TDV Sanofi-Pasteur). Large studies in Asia and Latin America demonstrated overall vaccine efficacy (VE) ranging from 44.6 to 65.6%, according to serotype, age of the recipient, and baseline flaviviral immunity [9]. However, concern remains that vaccine “escape” for certain serotypes/genotypes could occur and recently vaccination programs have been suspended due to emerging evidence of enhanced disease in flaviviral naïve vaccinees [9, 10•]. There is urgent need for improved real-time molecular characterization of dengue viruses.

The clinical presentations of dengue are broad, ranging from a mild febrile illness to life-threatening manifestations of organ impairment, hemorrhagic tendencies, and capillary leakage leading to hypovolemic shock. In 2009, the WHO reclassified dengue into dengue with and without warning signs and severe dengue [11]. The set of “warning signs” is intended to help clinicians identify patients likely to develop complications. Patients with any of the warning signs or patients deemed to be at higher risk are usually monitored as inpatients, and during seasonal epidemics, health care facilities can quickly become overwhelmed. There are currently no licensed therapeutics for dengue, and management relies on supportive treatment with cautious fluid replacement for those identified to have significant plasma leakage. Improved monitoring of patients, utilizing non-invasive and novel technologies would be a major advance, not only for better individual case management but also for improved utilization of resources, allowing more outpatient-based care.

The focus of this review is to define the unmet needs and challenges in current dengue diagnostics and clinical patient monitoring, and to identify new innovative technologies, with the aim of impacting on surveillance, outbreak response, and patient outcomes on individual and population levels.

## Clinical Manifestations

The continuum of symptoms for dengue infections ranges from mild self-limiting illness to life-threatening disease [12]. DENV infection has three phases, an initial febrile phase, lasting 1–4 days, a critical phase usually around days 4–6, and a recovery phase from days 7–10. Patients presenting with dengue in the febrile phase have fairly non-specific symptoms with an acute high-grade fever and constitutional symptoms (nausea, vomiting, rash, muscle aches, and headache) [12]. Other diseases such as malaria, chikungunya, and Zika may be endemic in the same region, and can also present similarly, however distinguishing these diseases early is important as all require differing management.

The critical phase, which lasts for roughly 48 h around defervescence, is the period where the increase in capillary permeability resulting in plasma leakage becomes apparent. This may present clinically as pleural effusions and/or ascites depending on the degree of plasma leakage [13]. Other markers include hypoalbuminemia and a rise in the hematocrit, and once a certain plasma volume is lost, cardiovascular collapse and shock follow. Dengue shock syndrome is defined by a pulse pressure of < 20 mmHg, with signs of impaired peripheral perfusion, including rapid weak pulse. This phenomenon of narrowing of the pulse pressure, whereby the diastolic blood pressure rises towards the systolic pressure, is fairly unique to dengue, and is thought to be due to slow and persistent plasma leakage occurring over several days that allows time for compensatory mechanisms to take place [14, 15]. Increased diastolic pressure is likely to occur secondary to a high systemic vascular resistance, and shutting down of peripheral vascular networks, to preserve perfusion to critical organs in the face of gradually worsening hypovolemia [16].

Although the critical period is when the plasma leakage becomes clinically apparent, the onset of the increase in capillary permeability starts in the febrile phase and subclinical fluid accumulation likely occurs to some degree in most dengue cases [17, 18]. The platelet count falls over the course of the infection reaching a nadir around defervescence. A sharp fall in platelets along with a rise in hematocrit has been included as one of the WHO dengue warning signs, with the recommendation that these patients require more intensive monitoring. Other warning signs that may precede severe dengue include abdominal pain, persistent vomiting, clinical fluid accumulation, mucosal bleed, lethargy/restlessness, and hepatomegaly.

The critical period lasts for 24–48 h after which the capillary leakage resolves and the extravascular fluid begins to be resorbed over the next 48–72 h. This period is known as the recovery phase. If intravenous fluids are continued into this period, there is significant risk of fluid overload, manifesting as respiratory distress from pleural effusions, pulmonary edema, and/or ascites.

While the majority of dengue patients experience a relatively benign disease course, detecting those who will develop significant complications currently remains challenging [19]. However, as the severe manifestations occur relatively late in the disease course, there is a window of opportunity to potentially identify patients who may progress, and where novel technology could be used to predict outcome in dengue.

## Challenges and Unmet Needs

### Current Diagnostics

Various technologies for dengue diagnosis have been developed, ranging from nucleic acid amplification tests (NAAT), to serology tests for antigen, enzyme-linked immunosorbent assay (ELISA), or plaque reduction and neutralization tests (PRNT) for antibodies and immunochromatography (Tables 1 and 2) [20–28]. Current diagnostics methods and strategies for dengue vary in sensitivity depending on a number of host and viral factors. In the first week of the acute infection, the gold standard is molecular techniques to isolate DENV RNA, using RT-PCR or detection of the non-structural protein 1 (NS1) using either ELISA or rapid antigen test [29]. NS1 protein, a highly conserved glycoprotein produced along with replication of DENV, and NS1 tests are widely used in clinical settings [30, 31]. However, as the viremia is short lived, if patients present after the end of the first week, confirmation relies on serodiagnostic tests, with seroconversion of IgM or at least a fourfold increase in IgG antibody titers in paired acute and convalescent serum specimens.

**Table 1:** Summary of dengue diagnostic tests for DENV IgM, IgG, and NS1 antigens

Product	Manufacturer, country	Method	Analytes	Sample type	Sample volume (uL)	Sensitivity (%)	Specificity (%)	Time (min)	Storage (°C)
Panbio Dengue Early Rapid Kit	Alere, USA	LF	NS1 Ag	S	50	58.6–91.9	92.5–98.4	15	2–8
Panbio Dengue Duo Cassette	Alere, USA	LF	IgM/IgG	S/P/WB	10	70.7–98.8	80.0–91.6	15	2–30
Panbio Dengue Early ELISA	Alere, USA	ELISA	NS1 Ag	S	75	71.7–83.6	82.5–98.74	130	2–8
Panbio Dengue IgG Capture ELISA	Alere, USA	ELISA	IgG	S	100	73.8–96.3	76.9–100	130	2–8
Panbio Dengue IgM Capture ELISA	Alere, USA	ELISA	IgM	S	100	46.6–94.7	85.4–100	130	2–8
SD Bioline Dengue Duo	Alere, USA	LF	NS1 Ag/IgM/IgG	S/P/WB	105	53.5–79.2	89.4–100	15–20	RT
ImmunoComb II Dengue IgM & IgG Bispot	Alere, USA	EIA	IgM/IgG	S/P	10	84–95	82	150	2–8
Dengue NS1 Ag Strip	Bio-Rad, France	W	NS1 Ag	S/P	50	79.1–92.3	98.8–100	15–30	2–8
Platelia Dengue NS1 Ag	Bio-Rad, France	EIA	NS1 Ag	S/P	50	69–97	99–100	140	2–8
IMMUNOQuick Dengue IgG/IgM	Biosynex, France	W	IgM/IgG	S/P/WB	1	95.2–97.6	96.6–98.3	10–20	2–30
OnSite Dengue Ag (NS1) Rapid Test	CTK Biotech, USA	LF	NS1 Ag	S/P/WB	90–105	100	100	20–25	2–30
OnSite Dengue IgG/IgM Combo Rapid Test	CTK Biotech, USA	LF	IgM/IgG	S/P/WB	30–50	91.6–96.1	96.5–97.5	20–25	2–30
OnSite Duo Dengue Ag-IgG/IgM	CTK Biotech, USA	LF	NS1 Ag/IgM/IgG	S/P/WB	5–100	96	97.3	20–25	2–30
RecombiLISA Dengue IgM ELISA Kit	CTK Biotech, USA	ELISA	IgM	S/P/WB	100	90.8–96.6	95.9–98.1	125	2–8
RecombiLISA Dengue IgG ELISA Kit	CTK Biotech, USA	ELISA	IgG	S/P	100	96.4	98.1	70	2–8
RecombiLISA Dengue Ag ELISA Kit	CTK Biotech, USA	ELISA	NS1 Ag	S/P	50	100	97.8	150	2–8
DENV Detect IgM Capture ELISA	InBios, USA	ELISA	IgM	S	50	91.7–97.4	92.8–100	110	2–8
Dengue NS1 Detect Rapid Test	InBios, USA	LF	NS1 Ag	S	50	76.5	97.4	30	RT
Dengue Fever IgG and IgM combo Device	Merlin, USA	LF	IgM/IgG	S/P/WB	1	96–97	98	30	2–30
ASSURE	MP Diagnostics, USA	LF	IgA	S/P/WB	25	86.7–99.4	86.1–99.2	20	2–28
Pathozyme dengue IgM and IgG	Omega Diagnostics, UK	ELISA	IgM	S	100	99.1	96.9	150	2–8
ImmunoComb II Dengue IgM & IgG Bispot	Orgenics	LF	IgM	S/P	10	95	82	150	2–8
Dengucheck WB	Zephyr Biomedicals, India	LF	IgM/IgG	S/P/WB	5	100	100	15	4–30

This table displays information as stated in company websites, product specification sheet, or references (Supplementary material). Sensitivity varies depending on dengue infection stage (acute vs convalescent) and primary vs secondary infections. This table does not intend to cover all commercially available dengue diagnostic tests for DENV IgM, IgG, and NS1 antigens

*W*, wick style; *LF*, lateral flow; *ELISA*, enzyme-linked immunosorbent assay; *EIA*, enzyme immune assay; *S*, serum; *P*, plasma; *WB*, whole blood; *RT*, room temperature

**Table 2:** Summary of molecular tests for dengue diagnosis

Product	Manufacturer, country	Method	Genotyping	Quantitative*
Geno-Sen’s DENGUE 1–4 Real time PCR kit	Genome Diagnostics, India	Real-time RT-PCR (TaqMan)	No	Yes
Dengue Virus General-type Real Time RT-PCR Kit	Shanghai ZJ Bio-Tech, China	Real-time RT-PCR (TaqMan)	No	Yes
RealStar Dengue RT-PCR kit 1.0	Altona Diagnostics, Germany	Real-time RT-PCR (TaqMan)	No	No
Simplexa Dengue Kit	Focus Diagnostics, USA	Real-time RT-PCR (TaqMan)	Yes	No
abTES DEN 5 qPCR I Kit	AITbiotech, Singapore	Real-time RT-PCR	Yes	No
DENV-specific TwistAmp RT exo lyophilized kit	TwistDx, UK	RT-RPA	No	No
DiaPlexQ ZCD Virus Detection Kit	SolGent Co, Republic of Korea	Real-time RT-PCR	No	No
FTD Zika/Dengue/Chik assay	Fast-Track Diagnostics, Malta	Real-time RT-PCR (TaqMan)	No	No
VIASURE Zika, Dengue & Chikungunya Real Time PCR Detection Kit	Certest Biotec, Spain	Real-time RT-PCR	No	No
AccuPower ZIKV (DENV, CHIKV) Multiplex Real-Time RT-PCR Kit	Bioneer, Republic of Korea	Real-time RT-PCR	No	No
Trioplex Real-time RT-PCR	Thermo Fisher Scientific, USA	Real-time RT-PCR (TaqMan)	No	No

Please note that this table does not intend to cover all commercially available molecular tests for dengue diagnosis. This table displays information as stated in company websites, product specification sheet, or references (Supplementary material)

*RT-PCR*, reverse transcriptase PCR; *RT-RPA*, reverse transcriptase recombinase polymerase amplification

*Real-time PCR assays can be used quantitatively, however this may not always be stated on the company website or product specification

However, there are several limitations with these current techniques. First, in addition to the short detection window by molecular techniques in early disease, these require a processing time of a few hours, as well as expensive and bulky equipment, and have varying lower limits of detection [32]. There are various commercial kits using ELISA and lateral flow tests to detect NS1 tests often paired with IgM/IgG, however they have variable sensitivity due to fluctuation of NS1 antigenemia depending on serotype and baseline immunity [23, 33, 34]. Secondary DENV infections generally have a shorter duration of NS1 antigenemia than primary infections, limiting its utility in these patients [35]. Serological tests face even more challenges, due to the cross-reactivity of antibodies to other flavivirus. Particularly in recent years, with the Zika virus pandemic, serodiagnosis has had limited use [36]. More sensitive ELISAs using algorithms to distinguish dengue from Zika IgM and IgGs are being developed, and commercial applications are awaited [37].

Overall, current methods used for diagnosing dengue needs improvement. An ideal dengue diagnostic should include the following specifications: (1) able to distinguish between dengue and other flavivirus, particularly those with similar presentation; (2) be highly sensitive, serotype specific, with a lower limit of detection in the range of 10 to 100 copies per assay; (3) be rapid or point-of-care (POC); and (4) has the potential for wireless connection to smartphones or the cloud, enabling real-time surveillance and rapid response to global epidemics. In addition, an ideal test for rural endemic areas should also be portable, inexpensive, stable to use in the field, little need for storage, and easy and safe to dispose [27].

### Current Clinical Monitoring

Accurate recognition of phase and severity of DENV infection allows proper placement of care, allowing dengue patients without warning signs to be managed in outpatient settings, patients with warning signs to be admitted for close monitoring, and those with signs of severe disease to be managed in facilities with access to intensive care if necessary.

Clinical identification of vascular leakage is challenging until or unless shock develops. Current techniques to identify and monitor plasma leakage rely on surrogate markers of intravascular volume depletion including serial hematocrit measurements and close observation of cardiovascular indices, in particular the pulse pressure and heart rate [38]. The identification of relative hemoconcentration is done by tracking changes in serial hematocrit measurements, with a rise of more than 20% from baseline considered evidence of significant leakage [39]. Regular POC hematocrits are also used during treatment of dengue shock patients to assess efficacy of fluid resuscitation, as outlined in the WHO guidelines [12]. Other POC test parameters that have been assessed for clinical monitoring include venous lactate measurements, which can be a surrogate for tissue perfusion [40, 41]. However, these tests require serial finger prick blood tests, which are uncomfortable and distressing particularly for younger patients.

Patients who develop profound shock or recurrent episodes of shock are monitored using invasive measures, involving the use of arterial lines and central venous pressure monitoring. However, in the setting of severe thrombocytopenia and coagulopathy, these invasive measures carry a high risk of bleeding complications, and there is an urgent need for non-invasive clinical monitors in dengue.

Our group has trialed other techniques of non-invasive hemodynamic assessment in severe dengue, including portable echocardiograms. Focused bedside echocardiography can be used for volume assessment and monitoring response to fluid administration, in addition to cardiac functional examination. This study showed that on day 1 of ICU admission, patients with echo-derived signs of severe intravascular volume depletion were more likely to get recurrent episodes of shock and receive the most intravenous fluids and develop respiratory distress [40].

Although portable echocardiography is a good way of assessing volume status non-invasively, and new handheld devices are being developed, it still requires training and expertise of staff, as well as expensive equipment and is also labor intensive. Novel technology for hemodynamic and intravascular volume assessment of dengue patients should be simple to use, completely non-invasive, cheap, and wearable with ideally wireless connectivity to allow remote monitoring—thus allowing widespread application for both hospitalized and ambulatory patients and appropriate for low–middle-income settings.

## Innovative Technologies

### Diagnostics

In addition to RT-PCR assays available for DENV detection and typing, there are other isothermal molecular technologies that have been recently developed. Isothermal amplification methods do not rely on thermal cycling and have emerged as a promising alternative to PCR that significantly simplifies the implementation of nucleic acid-based methods in POC diagnostic devices [42•, 43]. One of these is nucleic acid sequence-based amplification (NASBA), which amplifies RNA from a RNA target at 41 °C using a T7 RNA polymerase [44]. The amplicons produced by NASBA are detected in real-time by molecular beacons. None of the DENV NASBA assays have been commercialized yet, but they have the potential for affordable POC applications. Another isothermal method that has been developed for DENV detection and typing is the RT loop-mediated isothermal amplification (RT-LAMP), which uses strand displacement and amplification of its stem loop structure typically at 63 °C [45]. The LAMP chemistry is well suited for on-field applications since detection of the amplified products can be observed by measurement of fluorescence [46], magnesium pyrophosphate [47], electrochemical [48] colorimetric signal changes, detectable by the naked human eye [49], or unmodified cellphone cameras [43]. The DENV RT-LAMP is simple and rapid (results within 30 min) with no cross-reactivity and able to both detect and discriminate serotypes. Recent work from Imperial College London has shown significant promise of using RT-LAMP with semiconductor microchip technology through ion-sensitive field effect transistor (ISFET) sensing arrays [48, 50, 51•], enabling a future low-cost portable solution for DENV serotyping connected to a smartphone for field-based diagnosis in developing countries [52•]. Transcription-mediated amplification (TMA) is another isothermal method that has been developed for DENV detection [53], although as yet, it cannot be used for serotyping. TMA assay is based on the combination of two enzymes, T7 RNA polymerase and reverse transcriptase, to rapidly amplify the target DNA or RNA at a single temperature, around 62 °C. Molecular methods using TMA for detection of DENV infection have not yet been commercialized. The RT recombinase polymerase amplification (RT-RPA) has also been developed for DENV detection. The RT-RPA operates best at temperatures of 37–42 °C and is an excellent candidate for developing low-cost, rapid, point-of-care molecular tests. This isothermal chemistry uses recombinases to guide primers to target specific nucleic acid sites. Exploiting the speed (3–5 min, including the reverse transcriptase), sensitivity, and stability of RT-RPA, a research team in Germany has developed a fully mobile suitcase lab platform that can be used for the field-based diagnosis of DENV [54].

The aforementioned molecular methods, NASBA, RT-LAMP, TMA, and RT-RPA, are promising and more appropriate than RT-PCR for near-patient DENV testing, but these methods still require further development and validation before clinical deployment.

There are also a number of NAAT-based platforms in the pipeline that have the potential to be used for the diagnosis of acute DENV infection, but to date, only the Truelab Real-Time micro PCR System (Molbio Diagnostics) is commercially available. Other manufacturers have not yet developed DENV assays for their platforms, such as the GeneXpert Omni Platform (Cepheid), the Alere-i Platform (Alere Inc), or the cobas® Liat Analyser (Roche Molecular Diagnostics), but as the dengue pandemic increases, this may change in the near future.

## Sensors and Wearable Monitors

Sensors that measure the body’s vital signs, including blood pressure, heart rate, respiratory rate, blood oxygen saturation, and core temperature, have been around for many years and are used every day in all health care settings. In hospitalized patients, vital signs are commonly measured in both general wards and in intensive care settings to assess the condition of the patient and to identify clinical deterioration. Recent advances in wireless communication, low-power microelectronics [55], sensor manufacturing [56], and data analysis techniques [57] have opened up new possibilities for using wearable technology in the digital health ecosystem. As technology matures, and wearables and sensors are further miniaturized, more novel and connected applications for health care are being developed. The integration of medical sensors into consumer electronics enables home-based medical data gathering and supports remote care and preventive digital health programs. There are many examples of wearable sensors for health monitoring, and although some devices are already in the market, few examples are actually found in health care settings [58•]. In the context of the clinical management of dengue, non-invasive, continuous measurement of several routine clinical parameters, including vital signs and hematocrit would be of huge benefit, and existing wearable technologies would need to be encouraged to do so.

## Conclusion and Future Application

The widespread application of these novel platforms for POC dengue diagnostics, as well as wearable patient monitors, has the potential to revolutionize dengue surveillance and management at population and individual levels. The ability to diagnose dengue accurately and rapidly to the level of serotype and even genotype in both central and rural facilities has enormous potential, not only by tailoring individual patient care, in the form of targeted dengue management and potentially avoiding unnecessary antibiotic prescriptions, but also at the community level, by informing serotype/genotype predominance. This data collection and integration could facilitate an early warning system and epidemic potential, and allow planning of local health resources and activation of public health measures.

Wearable patient monitors could also impact greatly on dengue management in endemic areas; by safely keeping dengue patients as outpatients, this would improve health resource allocation and associated cost savings, which in large outbreaks could translate to freeing up large numbers of hospital beds and allowing more focused care on the severe cases. Wearable monitors could also allow earlier risk prediction for severe dengue by continuously measuring physiological parameters associated with vascular leak; patients can be identified earlier, allowing targeted supportive measures to be given prior to cardiovascular decompensation.

In summary, new developments in biotechnology are transforming all fields of medicine, and the benefits of these innovative technologies now urgently need to be applied to neglected tropical diseases like dengue and in resource-limited settings, as a global health priority.

## Electronic supplementary material

ESM 1 (DOCX 16.3 kb)
