# Supplementary material for: Improving Dengue Diagnostics and Management Through Innovative Technology
Source: Curr Infect Dis Rep. 2018 Jun 7;20(8):25. doi: 10.1007/s11908-018-0633-x (PMC5992235; doi:10.1007/s11908-018-0633-x)
Supplement: Supplementary file 1 — (DOCX 16.3 kb) [file 11908_2018_633_MOESM1_ESM.docx]

**Table 1**

1. <http://www.lyherbio.com/uploads/201712759/r201706261725136352044.pdf>
2. <https://www.sekisuidiagnostics.com/writable/product_documents/files/ifu_01pe40_dengue_early_elisa_2013.pdf>
3. <https://www.alere.com/en/home/product-details/panbio-dengue-igg-capture-elisa-au.html>
4. <http://www.biodiagnosticos.com/fichas-tecnicas/pruebas-infecciosas/panbio/dengue-igg-elisa-de-captura.pdf>
5. <https://www.sekisuidiagnostics.com/writable/product_documents/files/ifu_01pe20_dengue_igm_capture_2013.pdf>
6. <https://www.alere.com/en/home/product-details/panbio-dengue-igm-capture-elisa.html>
7. <https://www.sekisuidiagnostics.com/writable/product_documents/files/ifu_01pe20_dengue_igm_capture_2013.pdf>
8. <http://proton.com.ro/image/catalog/catalog_pdf/BIOSYNEX.pdf>
9. <http://athenesedx.com/insert/R0063C.pdf>
10. <http://www.harmony-vos.sk/ORGENICS/PRIBALOVE/RAPID/R0061C%20DENGUE%20.pdf>
11. <http://epimed.com.br/anexos/Dengue%20Duo%20Novo.pdf>
12. <http://ctkbiotech.com/ctk-product/dengue-igm-elisa-test-ce/>
13. <http://ctkbiotech.com/ctk-product/dengue-igg-elisa-kit-test-ce/>
14. <http://ctkbiotech.com/ctk-product/dengue-ag-elisa-test-ce/>
15. <http://athenesedx.com/insert/E0310.pdf>
16. <http://athenesedx.com/insert/E0312.pdf>
17. <http://www.athenesedx.com/marketing/dengue.pdf>
18. <http://www.tokyofuturestyle.com/wp/wp-content/uploads/900111-00-RUO-Dengue-NS1-Detect-Rapid-Test-Dry-printable-version.pdf>
19. <http://www.omegadiagnostics.com/portals/0/spanish-od647.pdf>
20. <http://www.tulipgroup.com/Zephyr_New/html/pack_inserts/Denguchek%20WB.pdf>
21. D Smith M, Azizan A. Current global status of dengue diagnostics. Journal of Advances in Biology & Biotechnology. 2015;2(2): 79-95.
22. Hunsperger EA, Yoksan S, Buchy P, Nguyen VC, Sekaran SD, Enria DA, Pelegrino JL, Vázquez S, Artsob H, Drebot M, Gubler DJ. Evaluation of commercially available anti–dengue virus immunoglobulin M tests. Emerging infectious diseases. 2009;15(3):436.
23. <http://www.who.int/tdr/publications/documents/diagnostics-evaluation-3.pdf>
24. <http://www.idc-dx.org/resources/dengue-virus-infection-diagnostics-landscape>

**Table 2**

1. Najioullah F, Viron F, Césaire R. Evaluation of four commercial real-time RT-PCR kits for the detection of dengue viruses in clinical samples. Virology journal. 2014;11(1):164.
2. <http://www.aitbiotech.com/wp-content/uploads/AITbiotech-abTES-MDx-Product-List_2016.pdf>
3. Saengsawang J, Nathalang O, Kamonsil M, Watanaveeradej V. Comparison of two commercial real-time PCR assays for detection of dengue virus in patient serum samples. Journal of clinical microbiology. 2014;52(10):3781-3.
4. Teoh BT, Sam SS, Tan KK, Danlami MB, Shu MH, Johari J, Hooi PS, Brooks D, Piepenburg O, Nentwich O, Wilder-Smith A. Early detection of dengue virus by use of reverse transcription-recombinase polymerase amplification. Journal of clinical microbiology. 2015;53(3):830-7.
5. http://www.solgent.com/eng/item_sub_list2.php?cate1_no=1&cate2_no=5&cate3_no=0&cate4_no=0&cate_check=0
6. http://www.fast-trackdiagnostics.com/human-line/products/ftd-zikadenguechik/
7. http://www.certest.es/products/zika-dengue-chikungunya-qpcr/
8. <http://eng.bioneer.com/diagnostic/humanmdxkits/Accupower-ZIKV-Multiplex-overview.aspx>
9. <https://www.fda.gov/downloads/medicaldevices/safety/emergencysituations/ucm491592.pdf>
